# Signatures of Altered Gene Expression in Dorsal Root Ganglia of a Fabry Disease Mouse Model

**DOI:** 10.3389/fnmol.2017.00449

**Published:** 2018-01-25

**Authors:** Kai K. Kummer, Theodora Kalpachidou, Michaela Kress, Michiel Langeslag

**Affiliations:** Division of Physiology, Department of Physiology and Medical Physics, Medical University of Innsbruck, Innsbruck, Austria

**Keywords:** Fabry disease, alpha Galactosidase A, lysosomal storage disorder, neuropathy, neurodegeneration, neuropathic pain

## Abstract

Fabry disease is an X-linked lysosomal storage disorder with involvement of the nervous system. Accumulation of glycosphingolipids within peripheral nerves and/or dorsal root ganglia results in pain due to small-fiber neuropathy, which affects the majority of patients already in early childhood. The α-galactosidase A deficient mouse proved to be an adequate model for Fabry disease, as it shares many symptoms including altered temperature sensitivity and pain perception. To characterize the signatures of gene expression that might underlie Fabry disease-associated sensory deficits and pain, we performed one-color based hybridization microarray expression profiling of DRG explants from adult α-galactosidase A deficient mice and age-matched wildtype controls. Protein-protein interaction (PPI) and pathway analyses were performed for differentially regulated mRNAs. We found 812 differentially expressed genes between adult α-galactosidase A deficient mice and age-matched wildtype controls, 506 of them being upregulated, and 306 being downregulated. Among the enriched pathways and processes, the disease-specific pathways “lysosome” and “ceramide metabolic process” were identified, enhancing reliability of the current analysis. Novel pathways that we identified include “G-protein coupled receptor signaling” and “retrograde transport” for the upregulated genes. From the analysis of downregulated genes, immune-related pathways, autoimmune, and infection pathways emerged. The current analysis is the first to present a differential gene expression profile of DRGs from α-galactosidase A deficient mice, thereby providing knowledge on possible mechanisms underlying neuropathic pain related symptoms in Fabry patients. Therefore, the presented data provide new insights into the development of the pain phenotype and might lead to new treatment strategies.

## Introduction

Fabry disease (FD) is an X-linked lysosomal storage disorder with estimated incidence rates of 1:37,000 for the classical Fabry phenotype and 1:3,100 for a late-onset disease variant (Spada et al., 2006; Mechtler et al., 2012). It can be caused by more than 500 different mutations of the lysosomal α-galactosidase A (α-Gal A) gene (Gal et al., 2006; Saito et al., 2011). Those mutations lead to deficient activity, reduction or depletion of α-Gal A, followed by impaired degradation of glycosphingolipids and subsequent accumulation of globotriaosylceramide (Gb3) in a variety of tissues, including vascular endothelial cells and neurons (Desnick et al., 2001; Bangari et al., 2015). In general, males are more affected by the α-Gal A mutations, but also heterozygote females have a significant risk for major organ involvement due to random X-inactivation causing variable expression of α-Gal A and decreased quality of life (Wilcox et al., 2008). One of the earliest symptoms of FD is pain due to small-fiber neuropathy, which affects the majority of patients already in early childhood. It can manifest as episodic crises with pain attacks originating in the extremities that can last for several days or even weeks, or chronic pain characterized by burning and tingling paraesthesia (Germain, 2010; Ginsberg, 2013). The origin of this pain phenotype presumably lies in accumulation of glycolipids within peripheral nerves and/or dorsal root ganglion (DRG) somata that might lead to degeneration of small sensory fibers (Kocen and Thomas, 1970; Ohnishi and Dyck, 1974; Bangari et al., 2015; Godel et al., 2017).

Investigating FD specific pathogenesis in Fabry patients is difficult and limited to molecular analyses of tissue biopsies and clinical neurophysiology techniques. It has been found that both motor and sensory conduction velocities are decreased, whereas vibratory, cold, and heat thresholds are elevated in Fabry patients (Sheth and Swick, 1980; Dutsch et al., 2002; Uceyler et al., 2013; Namer et al., 2017). In addition, the proportion of mechano-responsive C-fibers is reduced in patients compared to healthy controls (Namer et al., 2017). To investigate the molecular and physiological mechanisms underlying the pathology of FD, α-galactosidase A deficient mice [α-Gal A^(−/0)^] were generated which share many symptoms with Fabry patients, including altered temperature sensitivity and pain perception (Ohshima et al., 1997; Lakoma et al., 2014; Uceyler et al., 2016; Namer et al., 2017).

Although FD constitutes a monogenic disease with loss of function mutations of the α-Gal A gene causing the disease, other genes and/or gene products might be indirectly regulated during disease progression and could play important roles in the manifestation of disease-specific pathologies and symptoms, like the development of small-fiber neuropathy. In the current study we therefore performed mRNA microarray expression profiling of DRG samples from α-Gal A^(−/0)^ mice aged > 20 weeks when the disease is fully developed to investigate the mRNA signatures associated with FD peripheral nerve neuropathy.

## Methods

### Animals

Male α-galactosidase A^(−/0)^ (α-Gal A^(−/0)^; background C57BL/6; provided by Dr. A. Kulkarni, National Institute of Health, NIDCR, Bethesda, USA) (Ohshima et al., 1997) and wildtype C57BL/6J mice aged 20-24 weeks were inbred and housed under specific pathogen-free (SPF) conditions. For microarray expression profiling mice from the separate inbred colonies were used, whereas for RT-qPCR validation, α-Gal A^(−/0)^ mice backcrossed with wildtype C57BL/6J mice and wildtype C57BL/6J mice were used to control for inbred colony effects. Animals were maintained at constant room temperature of 24°C on a 12 h light/dark cycle with lights on from 07:00 to 19:00 and had *ad libitum* access to autoclaved pelleted food and water. All animals were treated in accordance with the Ethics Guidelines of Animal Care (Medical University of Innsbruck), as well as the European Communities Council Directive of 22 September 2010 on the protection of animals used for scientific purposes (2010/63/EU), and approved by the Austrian National Animal Experiment Ethics Committee of the Austrian Bundesministerium für Wissenschaft und Forschung (permit number BMWF-66.011/0054-WF/V/3b/2015).

### Tissue collection

For microarray expression profiling eight adult mice (aged between 20 and 24 weeks) per group, whereas for RT-qPCR validation six adult mice (aged between 20 and 24 weeks) per group, were deeply anesthetized with isoflurane and euthanized by decapitation. Spinal cords were removed, lumbar DRGs L3-L5 (containing the cell bodies of primary afferents that project into the hind paw) harvested and flash-frozen in liquid nitrogen. Samples were kept at −80°C until further processing. For microarray expression profiling, DRGs from two mice were pooled for the final tissue sample.

### Microarray expression profiling

Genome-wide expression profiling was carried out by IMGM Laboratories (Munich, Germany) using Agilent SurePrint G3 Mouse GE 8 × 60K Microarrays in combination with a one-color based hybridization protocol. Microarray signals were detected using the Agilent DNA Microarray Scanner.

Total RNA including small RNAs was isolated using the miRNeasy Mini Kit (Qiagen) according to the manufacturer's instructions and eluted in 40 μl RNase-free water. RNA concentration and purity was determined on a NanoDrop ND-1000 spectral photometer (Peqlab). Samples were analyzed using the RNA 6000 Nano LabChip Kit (Agilent Technologies) on a 2100 Bioanalyzer (Agilent Technologies). For mRNA analysis, total RNA samples were spiked with *in vitro* synthesized polyadenylated transcripts (One-Color RNA Spike-In Mix, Agilent Technologies), reverse transcribed into cDNA and then converted into Cyanine-3 labeled complementary RNA (Low Input Quick-Amp Labeling Kit One-Color, Agilent Technologies) according to the manufacturer's instructions. cRNA concentration, RNA absorbance ratio, and Cyanine-3 dye concentration were recorded using a NanoDrop ND-1000 UV-VIS spectral photometer, and quality of labeled cRNA was analyzed using the RNA 6000 Nano LabChip Kit (Agilent Technologies) on a 2100 Bioanalyzer (Agilent Technologies). Following cRNA clean-up and quantification, Cyanine-3-labeled cRNA samples were fragmented and prepared for one-color-based hybridization (Gene Expression Hybridization Kit, Agilent Technologies) and hybridized at 65°C for 17 h on Agilent SurePrint G3 Mouse GE 8 × 60K Microarrays. After hybridization, microarrays were washed with increasing stringency using Triton X-102 supplemented Gene Expression Wash Buffers (Agilent Technologies) followed by drying with acetonitrile (Sigma). Fluorescence signals were detected on an Agilent DNA Microarray Scanner and extracted using feature extraction software (Agilent Technologies). The data discussed in this publication have been deposited in NCBI's Gene Expression Omnibus (Edgar et al., 2002) and are accessible through GEO Series accession number GSE104625 (https://www.ncbi.nlm.nih.gov/geo/query/acc.cgi?acc=GSE104625).

### Bioinformatics analyses

GeneSpring GX 13.0 analysis software (Agilent Technologies) was used to normalize and analyze the microarray raw data. Data were normalized using non-parametric quantile normalization. Groups were compared using Welch's approximate *t*-test (unpaired unequal variances) and *p*-values corrected for multiple testing using the algorithm of Benjamini and Hochberg (Benjamini and Hochberg, 1995), controlling for false discovery rate (FDR). Differential expression between the two groups was determined by calculating fold changes of the averaged normalized expression values. Significantly regulated mRNAs were identified by applying filters on fold changes (absolute fold change ≥ 1.2 or ≥2) and *p*-values (*p* ≤ 0.01). Chip array data were further processed by *R statistics* statistical software package (R Development Core Team, 2008) and Volcano plots prepared using R statistics *ggplot function*. Only genes with uncompromised hybridization values in all individual samples were used for the current analysis.

### Protein-protein interaction analysis

Protein-protein interactions (PPIs) were investigated for the significantly regulated mRNAs using the STRING Database v. 10.5 (http://www.string-db.org) (Szklarczyk et al., 2017), which includes direct and indirect protein associations collected from different databases. Interaction networks were prepared using medium confidence scores (0.40) and clustered using MCL clustering algorithm (inflation parameter: 3). Disconnected nodes were hidden from the network.

### Functional enrichment and pathway analysis

Functional enrichment and pathway analyses were also performed using the STRING Database v. 10.5 (http://www.string-db.org). Classification systems tested were Gene Ontology and KEGG functional annotation spaces, employing Fisher's exact test followed by a correction for multiple testing (FDR). Only enriched pathways with FDR corrected *p* < 0.05 are reported.

### RT-qPCR validation of regulated genes

Reverse transcription quantitative polymerase chain reaction (RT-qPCR) validation of regulated genes was performed using TaqMan Gene Expression Assays (Thermo Fisher Scientific) in an Applied Biosystems 7500 Fast Real-Time PCR System (Thermo Fisher Scientific).

Total RNA was extracted using peqGOLD TriFast reagent (Peqlab) according to the manufacturer's instructions. The quality and quantity of RNA was evaluated using NanoDrop 2000 (Thermo Scientific). Reverse transcription of mRNA was performed as previously described (Langeslag et al., 2014). Genes of interest were analyzed by RT-qPCR using the following TaqMan Gene Expression Assays (Thermo Fisher Scientific): Mm00557794_m1 (Amz1), Mm00476032_m1 (Atf3), Mm01299527_m1 (Dnah8), Mm01311685_m1 (Dnase1l3), Mm00469610_m1 (Ecel1), Mm00521881_m1 (Meig1), Mm00505317_m1 (Ncapg2), Mm00443523_m1 (Opn4), Mm01279059_m1 (Rnf39), Mm00509406_m1 (Samd8), Mm00555659_m1 (Dock4), Mm03646971_gH (Gm1987), Mm02391771_g1 (Hdac1), Mm00440480_m1 (Nnat), Mm00452229_m1 (Pmepa1), Mm01188211_m1 (S100pbp), Mm00521530_m1 (Slc51a), Mm00628467_m1 (Syt15), Mm00503605_m1 (Tmem25), Mm00836474_m1 (Zfp932), Mm00446968_m1 (Hprt), Mm01352363_m1 (Sdha), and Mm00441941_m1 (Tfrc). Experimental procedures were performed according to the TaqMan Gene Expression Assays protocol. The reactions were loaded on MicroAmp Fast Optical 96-well reaction plates (Thermo Fisher Scientific) and placed in the Applied Biosystems 7500 Fast Real-Time PCR System (Thermo Fisher Scientific). The PCR cycle protocol used was: 10 min at 95°C, 40 two-step cycles of 15 s at 95°C and 1 min at 60°C. Each sample was run in duplicates alongside non-template controls. Threshold was set manually at 0.1 and threshold cycle (C_T_) was used as a measure of initial RNA input. Relative fold changes in gene expression were calculated using the 2^−ΔΔCT^ method. All fold changes were expressed relative to the respective expression in wildtype mice and analyzed using Welch's *t*-test. Three genes (i.e., Hprt, Sdha and Tfrc) were used as reference genes. All three reference genes were found to be stably expressed in both groups of animals, as indicated by geNorm, Normfinder, and Bestkeeper software packages.

## Results

### mRNA expression profile of fabry mouse dorsal root ganglia

**Figure 1:**
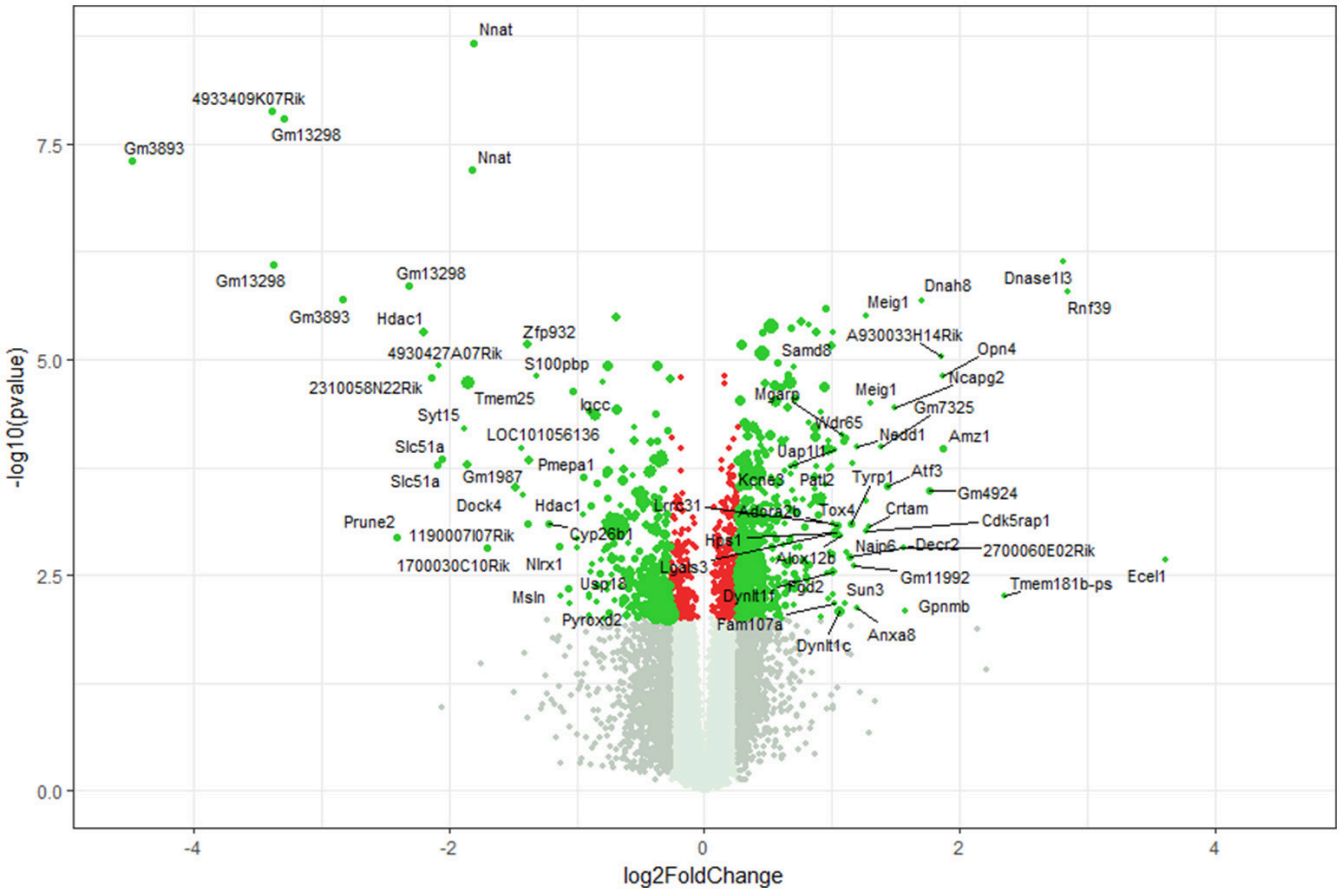
Volcano plot microarray data. Color green, *p* ≤ 0.01, fold change ≥ 1.2; labels, *p* ≤ 0.01, fold change ≥ 2.0; dot size represents relative expression values of wildtype mice.

**Figure 2:**
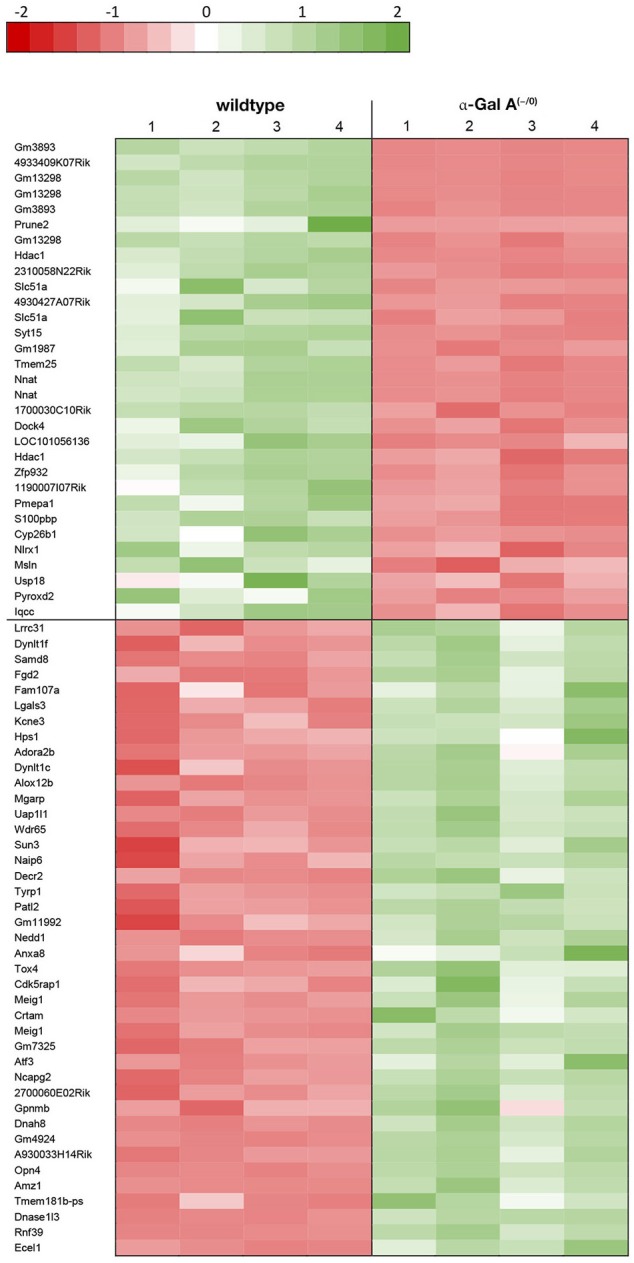
Heatmap of significantly regulated genes.

Using microarray expression profiling we found that in total 812 genes from the overall 21,736 detected mRNAs were significantly different between DRG samples from wildtype and α-Gal A^(−/0)^ mice (criteria *p* ≤ 0.01, absolute fold change ≥ 1.2) (Figure 1). Of those, 506 genes were significantly upregulated and 306 genes were significantly downregulated as compared to wildtype controls. More stringent filtering (criteria *p* ≤ 0.01, absolute fold change ≥ 2) of those significantly regulated genes revealed an assessable number of 78 genes in total (Figure 2). Using these criteria 41 genes were significantly upregulated, of which 29 showed FDR corrected *p* ≤ 0.1 (Table 1). Furthermore, 31 genes remained significantly downregulated, of which 27 showed FDR corrected *p* ≤ 0.1 (Table 2). PPI analysis (STRING Database) neither revealed clusters of interacting proteins nor enriched pathways, due to the small number of input genes. Thus, for in depth PPI analysis all significantly regulated genes (less stringent filtering, criteria *p* ≤ 0.01, absolute fold change ≥ 1.2) were used.

**Table 1:** Raw expression values, fold changes and statistical analysis for significantly *upregulated* genes.

**NCBI RefSeq ID**	**Gene symbol**	**Gene name**	**Expression α-Gal A^(−/0)^**	**Expression wildtype**	**Fold change**	***p*-value**	**FDR**
NM_001277925	Ecel1	Endothelin converting enzyme-like 1	3,723	282	12.3	0.0021	0.1166
NM_001099632	Rnf39	Ring finger protein 39	1,048	138	7.2	<0.0001	0.0038
NM_007870	Dnase1l3	Deoxyribonuclease 1-like 3	286	39	7.1	<0.0001	0.0028
BC096660	Tmem181b-ps	Transmembrane protein 181B, pseudogene	290	55	5.1	0.0054	0.1676
NM_173405	Amz1	Archaelysin family metallopeptidase 1	1,865	476	3.7	0.0001	0.0327
NM_013887	Opn4	Opsin 4 (melanopsin)	149	40	3.7	<0.0001	0.0117
XR_105403	A930033H14Rik	RIKEN cDNA A930033H14 gene	215	58	3.6	<0.0001	0.0089
DQ459435	Gm4924	Predicted gene 4924	2,969	811	3.4	0.0003	0.0548
NM_013811	Dnah8	Dynein, axonemal, heavy chain 8	552	162	3.3	<0.0001	0.0044
NM_053110	Gpnmb	Glycoprotein (transmembrane) nmb	446	143	3.0	0.0081	0.1909
NM_026528	2700060E02Rik	RIKEN cDNA 2700060E02 gene	242	79	3.0	0.0015	0.0992
NM_133762	Ncapg2	Non-SMC condensin II complex, subunit G2	641	216	2.8	<0.0001	0.0181
NM_007498	Atf3	Activating transcription factor 3	1,776	611	2.7	0.0003	0.0524
NM_001177470	Gm7325	Predicted gene 7325	845	304	2.6	0.0001	0.0324
NM_008579	Meig1	Meiosis expressed gene 1	712	272	2.5	<0.0001	0.0168
NM_019465	Crtam	cytotoxic and regulatory T cell molecule	102	41	2.4	0.0009	0.0784
NM_008579	Meig1	Meiosis expressed gene 1	287	114	2.4	<0.0001	0.0059
NM_025876	Cdk5rap1	CDK5 regulatory subunit associated protein 1	487	191	2.4	0.0010	0.0839
NM_023434	Tox4	TOX high mobility group box family member 4	480	191	2.4	0.0004	0.0609
NM_013473	Anxa8	Annexin A8	142	60	2.3	0.0076	0.1879
NM_008682	Nedd1	Neural precursor cell expressed, developmentally down-regulated gene 1	192	81	2.3	0.0001	0.0321
NM_001037928	Gm11992	Predicted gene 11992	172	74	2.3	0.0025	0.1233
NM_026251	Patl2	Protein associated with topoisomerase II homolog 2 (yeast)	226	97	2.2	0.0002	0.0392
NM_031202	Tyrp1	Tyrosinase-related protein 1	2,358	983	2.2	0.0008	0.0755
NM_011933	Decr2	2-4-dienoyl-Coenzyme A reductase 2, peroxisomal	3,844	1,597	2.2	0.0020	0.1141
NM_010871	Naip6	NLR family, apoptosis inhibitory protein 6	145	65	2.2	0.0017	0.1073
NM_177576	Sun3	Sad1 and UNC84 domain containing 3	109	50	2.2	0.0067	0.1815
NM_026789	Wdr65	WD repeat domain 65	1,675	726	2.1	0.0001	0.0290
NM_001033293	Uap1l1	UDP-N-acteylglucosamine pyrophosphorylase 1-like 1	3,662	1,579	2.1	0.0001	0.0291
NM_026358	Mgarp	Mitochondria localized glutamic acid rich protein	421	191	2.1	0.0001	0.0279
NM_009659	Alox12b	Arachidonate 12-lipoxygenase, 12R type	135	63	2.1	0.0011	0.0869
NM_001166630	Dynlt1c	Dynein light chain Tctex-type 1C	16,719	7,247	2.1	0.0081	0.1909
NM_007413	Adora2b	Adenosine A2b receptor	1,478	667	2.1	0.0008	0.0771
AK031397	Hps1	Hermansky-Pudlak syndrome 1 homolog (human)	156	74	2.0	0.0010	0.0850
NM_020574	Kcne3	Potassium voltage-gated channel, Isk-related subfamily, gene 3	159	76	2.0	0.0001	0.0332
NM_001145953	Lgals3	Lectin, galactose binding, soluble 3	19,457	8,721	2.0	0.0010	0.0850
NM_183187	Fam107a	Family with sequence similarity 107, member A	277	132	2.0	0.0067	0.1813
NM_013710	Fgd2	FYVE, RhoGEF and PH domain containing 2	314	149	2.0	0.0053	0.1650
NM_026283	Samd8	Sterile alpha motif domain containing 8	362	171	2.0	<0.0001	0.0065
NM_001199948	Dynlt1f	Dynein light chain Tctex-type 1F	12,240	5,496	2.0	0.0029	0.1333
XR_002334	Lrrc31	Leucine rich repeat containing 31	689	325	2.0	0.0008	0.0755

**Table 2:** Raw expression values, fold changes, and statistical analysis for significantly *downregulated* genes.

**NCBI RefSeq ID**	**Gene symbol**	**Gene name**	**Expression α-Gal A^(−/0)^**	**Expression wildtype**	**Fold change**	***p*-value**	**FDR**
NR_033506	Gm3893	Predicted gene 3893	74	1,582	−22.4	<0.0001	0.0005
NR_033123	4933409K07Rik	RIKEN cDNA 4933409K07 gene	134	1,332	−10.5	<0.0001	0.0002
NM_001085530	Gm13298	Predicted gene 13298	214	2,104	−10.4	<0.0001	0.0028
NM_001085530	Gm13298	Predicted gene 13298	217	1,989	−9.8	<0.0001	0.0002
NR_033506	Gm3893	Predicted gene 3893	92	628	−7.1	<0.0001	0.0044
AK046830	Prune2	Prune homolog 2 (Drosophila)	256	1,279	−5.3	0.0012	0.0905
NM_001085530	Gm13298	Predicted gene 13298	143	683	−5.0	<0.0001	0.0038
NM_008228	Hdac1	Histone deacetylase 1	577	2,465	−4.6	<0.0001	0.0065
AK009987	2310058N22Rik	RIKEN cDNA 2310058N22 gene	342	1,408	−4.4	<0.0001	0.0117
AK147155	Slc51a	Solute carrier family 51, alpha subunit	526	2,093	−4.3	0.0002	0.0400
NM_134041	4930427A07Rik	RIKEN cDNA 4930427A07 gene	74	303	−4.2	<0.0001	0.0102
NM_145932	Slc51a	Solute carrier family 51, alpha subunit	130	513	−4.1	0.0001	0.0376
NM_181529	Syt15	Synaptotagmin XV	56	201	−3.7	0.0001	0.0254
NM_001193667	Gm1987	Predicted gene 1987	981	3,294	−3.6	0.0002	0.0400
NM_027865	Tmem25	Transmembrane protein 25	5,609	18,317	−3.6	<0.0001	0.0120
NM_010923	Nnat	Neuronatin	185	620	−3.5	<0.0001	0.0005
NM_010923	Nnat	Neuronatin	233	773	−3.5	<0.0001	0.0001
NR_015521	1700030C10Rik	RIKEN cDNA 1700030C10 gene	150	466	−3.2	0.0015	0.1002
NM_172803	Dock4	Dedicator of cytokinesis 4	1,369	3,479	−2.8	0.0003	0.0541
XM_003945535	LOC101056136	Disks large homolog 5-like	51	135	−2.7	0.0001	0.0326
NM_008228	Hdac1	Histone deacetylase 1	159	407	−2.7	0.0004	0.0591
NM_145563	Zfp932	Zinc finger protein 932	1,101	2,666	−2.6	<0.0001	0.0076
NM_001135567	1190007I07Rik	RIKEN cDNA 1190007I07 gene	571	1,387	−2.6	0.0008	0.0763
NM_022995	Pmepa1	Prostate transmembrane protein, androgen induced 1	2,274	5,394	−2.6	0.0001	0.0378
AK139097	S100pbp	S100P binding protein	100	239	−2.5	<0.0001	0.0117
NM_175475	Cyp26b1	Cytochrome P450, family 26, subfamily b, polypeptide 1	583	1,264	−2.3	0.0008	0.0756
NM_178420	Nlrx1	NLR family member X1	383	793	−2.2	0.0015	0.0991
NM_018857	Msln	Mesothelin	81	172	−2.2	0.0054	0.1674
NM_011909	Usp18	Ubiquitin specific peptidase 18	226	451	−2.1	0.0045	0.1552
NM_029011	Pyroxd2	Pyridine nucleotide-disulphide oxidoreductase domain 2	57	115	−2.1	0.0066	0.1804
NM_198026	Iqcc	IQ motif containing C	556	1,068	−2.0	<0.0001	0.0130

### Enriched pathways and protein-protein interactions for upregulated mRNAs

Enrichment analysis of the 506 significantly upregulated genes revealed that a number of KEGG pathways and Gene Ontology processes were enriched, including the KEGG pathway “Lysosome” (KEGG:04142) and the biological process “Ceramide metabolic process” (GO:0006672), both known to constitute major hallmarks of FD pathogenesis (Table 3).

**Table 3:** Enrichment-analysis for *upregulated* mRNAs in α-Gal A^(−/0)^ vs. wildtype mice using gene ontology and KEGG pathway annotations.

**Pathway ID**	**Pathway description**	**Count in network**	**False discovery rate**
**KEGG PATHWAYS**			
04142	Lysosome	12	0.0011
05204	Chemical carcinogenesis	9	0.0073
00980	Metabolism of xenobiotics by cytochrome P450	7	0.0184
00480	Glutathione metabolism	6	0.0317
00511	Other glycan degradation	4	0.0231
**BIOLOGICAL PROCESSES (GO)**			
GO:0008150	Biological_process	254	0.0328
GO:0009987	Cellular process	242	0.0139
GO:0044763	Single-organism cellular process	204	0.0139
GO:0008152	Metabolic process	184	0.0328
GO:1901564	Organonitrogen compound metabolic process	46	0.0139
GO:0033993	Response to lipid	30	0.0462
GO:0006672	Ceramide metabolic process	8	0.0328
**CELLULAR COMPONENT (GO)**			
GO:0005575	Cellular_component	299	0.0004
GO:0005623	Cell	273	0.0004
GO:0044464	Cell part	273	0.0004
GO:0005622	Intracellular	256	0.0001
GO:0043226	Organelle	251	<0.0001
GO:0044424	Intracellular part	251	0.0001
GO:0043227	Membrane-bounded organelle	239	<0.0001
GO:0005737	Cytoplasm	228	<0.0001
GO:0043229	Intracellular organelle	222	0.0010
GO:0043231	Intracellular membrane-bounded organelle	208	0.0009
GO:0016020	Membrane	172	0.0053
GO:0044444	Cytoplasmic part	164	<0.0001
GO:0044422	Organelle part	151	0.0309
GO:0044425	Membrane part	134	0.0088
GO:0031224	Intrinsic component of membrane	116	0.0149
GO:0016021	Integral component of membrane	112	0.0211
GO:0005576	Extracellular region	98	0.0222
GO:0031982	Vesicle	97	0.0001
GO:0005886	Plasma membrane	96	0.0095
GO:0071944	Cell periphery	96	0.0186
GO:0031988	Membrane-bounded vesicle	94	0.0001
GO:0044421	Extracellular region part	91	0.0044
GO:0070062	Extracellular exosome	77	0.0004
GO:0031090	Organelle membrane	69	0.0260
GO:0044459	Plasma membrane part	57	0.0041
GO:0005829	Cytosol	49	0.0335
GO:0098805	Whole membrane	49	0.0378
GO:0005739	Mitochondrion	48	0.0222
GO:0031226	Intrinsic component of plasma membrane	33	0.0170
GO:0005887	Integral component of plasma membrane	30	0.0434
GO:0005773	Vacuole	25	0.0006
GO:0048471	Perinuclear region of cytoplasm	25	0.0041
GO:0005764	Lysosome	23	0.0004
GO:0042470	Melanosome	8	0.0309
GO:0030904	Retromer complex	4	0.0200
GO:0097422	Tubular endosome	3	0.0044
GO:1990622	CHOP-ATF3 complex	2	0.0186

*Whole genome was used as statistical background*.

**Figure 3:**
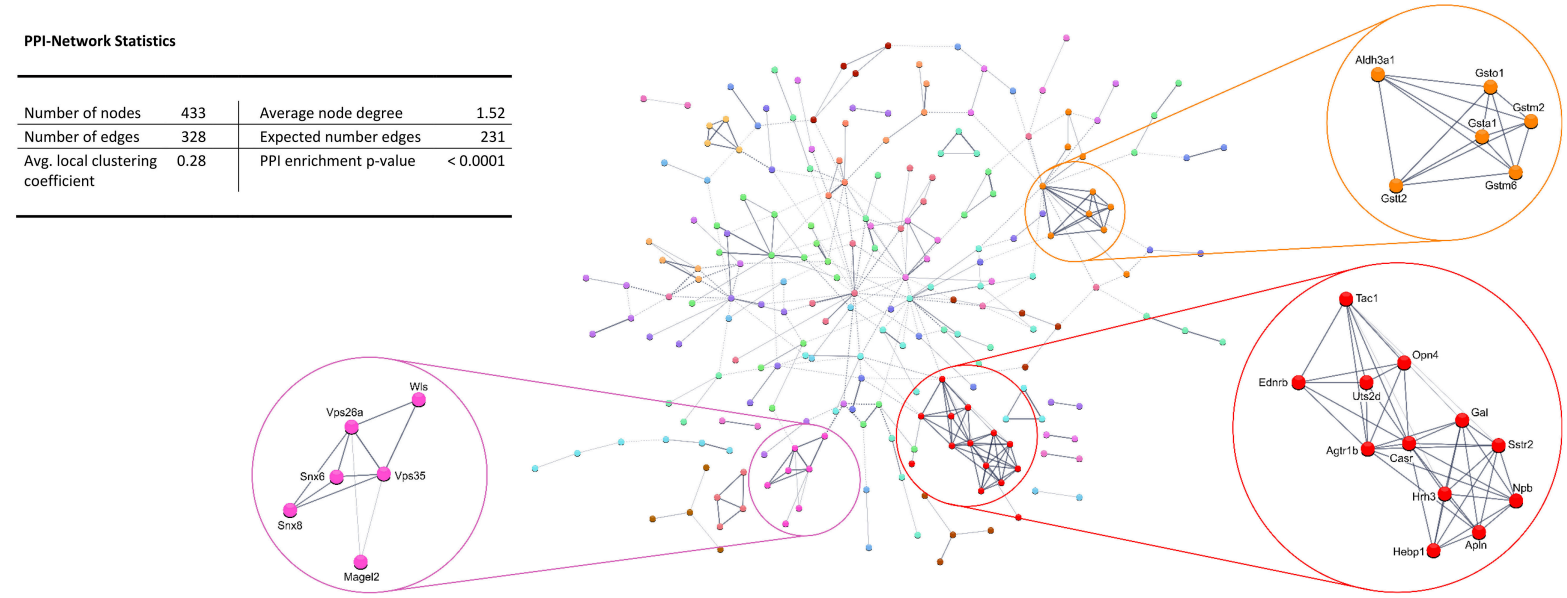
STRING database protein-protein interaction (PPI) network of significantly *upregulated* genes. Cut-off values, *p* ≤ 0.01, fold change ≥ 1.2.

Protein-protein interaction (PPI) analysis of significantly upregulated mRNAs revealed a significant PPI enrichment (*p* < 0.0001; Figure 3). The number of actually observed edges (*n* = 328) exceeded the expected number of edges (*n* = 231) by 42%. Furthermore, three clusters of at least five interconnected proteins became apparent. Enrichment analysis of those clusters showed that those genes were involved in different pathways (Table 4), the red cluster was related to “G-protein coupled receptor signaling” (e.g., GO:0007186), the pink cluster was involved in “retrograde transport” (GO:0042147) and the orange cluster was related to “glutathione transferase activity” (GO:0004364).

**Table 4:** GO biological processes and molecular functions of PPI-clusters from *upregulated* mRNAs in α-Gal A^(−/0)^ vs. wildtype mice.

**Pathway ID**	**Pathway description**	**Count in network**	**False discovery rate**
**RED CLUSTER**			
GO:0007186	G-protein coupled receptor signaling pathway	9	<0.0001
GO:0044057	Regulation of system process	7	<0.0001
GO:0004930	G-protein coupled receptor activity	6	0.0004
GO:0008217	Regulation of blood pressure	5	<0.0001
GO:0007218	Neuropeptide signaling pathway	4	0.0004
**PINK CLUSTER**			
GO:0042147	Retrograde transport, endosome to Golgi	5	<0.0001
**ORANGE CLUSTER**			
GO:0004364	Glutathione transferase activity	5	<0.0001

### Enriched pathways and protein-protein interactions for downregulated mRNAs

Enrichment analysis for the 306 significantly downregulated genes revealed a variety of regulated pathways, including immune related pathways (e.g., Complement and coagulation cascades, Antigen processing and presentation, Immune system process, Immune responses, etc.), autoimmune diseases (e.g., Systemic lupus erythematosus, Diabetes mellitus Type 1, Autoimmune thyroid disease, Asthma, etc.) and different infection pathways (e.g., Herpes simplex, Staphylococcus aureus, Leishmaniasis, etc.). In addition, “Neuroactive ligand-receptor interaction” (KEGG:04080) and “Vesicle” (GO:0031982) were enriched in the downregulated mRNAs (Table 5).

**Table 5:** Enrichment-analysis for *downregulated* mRNAs in α-Gal A^(−/0)^ vs. wildtype mice using gene ontology and KEGG pathway annotations.

**Pathway ID**	**Pathway description**	**Count in network**	**False discovery rate**
**KEGG PATHWAYS**			
05168	Herpes simplex infection	12	0.0003
04080	Neuroactive ligand-receptor interaction	10	0.0220
05164	Influenza A	9	0.0050
05322	Systemic lupus erythematosus	8	0.0007
05150	Staphylococcus aureus infection	7	0.0003
04514	Cell adhesion molecules (CAMs)	7	0.0247
04145	Phagosome	7	0.0319
05332	Graft-versus-host disease	6	0.0010
05330	Allograft rejection	5	0.0058
04940	Type I diabetes mellitus	5	0.0100
05320	Autoimmune thyroid disease	5	0.0159
05140	Leishmaniasis	5	0.0193
04612	Antigen processing and presentation	5	0.0220
05133	Pertussis	5	0.0220
05416	Viral myocarditis	5	0.0220
04610	Complement and coagulation cascades	5	0.0247
05310	Asthma	4	0.0050
04672	Intestinal immune network for IgA production	4	0.0220
**BIOLOGICAL PROCESSES (GO)**			
GO:0050896	Response to stimulus	93	<0.0001
GO:0044707	Single-multicellular organism process	75	0.0471
GO:0048523	Negative regulation of cellular process	61	0.0268
GO:0006950	Response to stress	54	0.0005
GO:0051239	Regulation of multicellular organismal process	43	0.0301
GO:0010033	Response to organic substance	38	0.0351
GO:0002376	Immune system process	36	<0.0001
GO:0006952	Defense response	31	0.0000
GO:0051240	Positive regulation of multicellular organismal process	30	0.0222
GO:0006955	Immune response	24	0.0007
GO:0007155	Cell adhesion	22	0.0465
GO:0045087	Innate immune response	19	0.0001
GO:0009607	Response to biotic stimulus	19	0.0280
GO:0051707	Response to other organism	18	<0.0001
GO:0098609	Cell-cell adhesion	17	0.0178
GO:0002252	Immune effector process	14	0.0080
GO:0016337	Single organismal cell-cell adhesion	14	0.0465
GO:0051962	Positive regulation of nervous system development	14	0.0465
GO:0034109	Homotypic cell-cell adhesion	12	0.0259
GO:0009615	Response to virus	10	0.0146
GO:0022409	Positive regulation of cell-cell adhesion	9	0.0396
GO:0019882	Antigen processing and presentation	7	0.0077
GO:0016064	Immunoglobulin mediated immune response	7	0.0080
GO:0006959	Humoral immune response	7	0.0280
GO:0002455	Humoral immune response mediated by circulating immunoglobulin	6	0.0015
GO:0048002	Antigen processing and presentation of peptide antigen	5	0.0280
GO:0019886	Antigen processing and presentation of exogenous peptide antigen via MHC class II	4	0.0080
GO:0070268	Cornification	2	0.0471
**CELLULAR COMPONENT (GO)**			
GO:0005575	Cellular_component	183	0.0002
GO:0044464	Cell part	160	0.0052
GO:0005623	Cell	160	0.0057
GO:0016020	Membrane	112	0.0004
GO:0044425	Membrane part	91	0.0003
GO:0031224	Intrinsic component of membrane	82	0.0002
GO:0016021	Integral component of membrane	80	0.0002
GO:0005886	Plasma membrane	68	0.0003
GO:0071944	Cell periphery	68	0.0005
GO:0005576	Extracellular region	62	0.0252
GO:0044421	Extracellular region part	58	0.0054
GO:0031982	Vesicle	57	0.0041
GO:0031988	Membrane-bounded vesicle	52	0.0252
GO:0070062	Extracellular exosome	49	0.0016
GO:0044459	Plasma membrane part	40	0.0011
GO:0005615	Extracellular space	25	0.0252
GO:0005887	Integral component of plasma membrane	24	0.0026
GO:0098797	Plasma membrane protein complex	16	0.0011
GO:0045121	Membrane raft	10	0.0252
GO:0072562	Blood microparticle	6	0.0252
GO:0042611	MHC protein complex	5	0.0002
GO:0042613	MHC class II protein complex	4	0.0001
GO:0035098	ESC/E(Z) complex	3	0.0252

*Whole genome was used as statistical background*.

**Figure 4:**
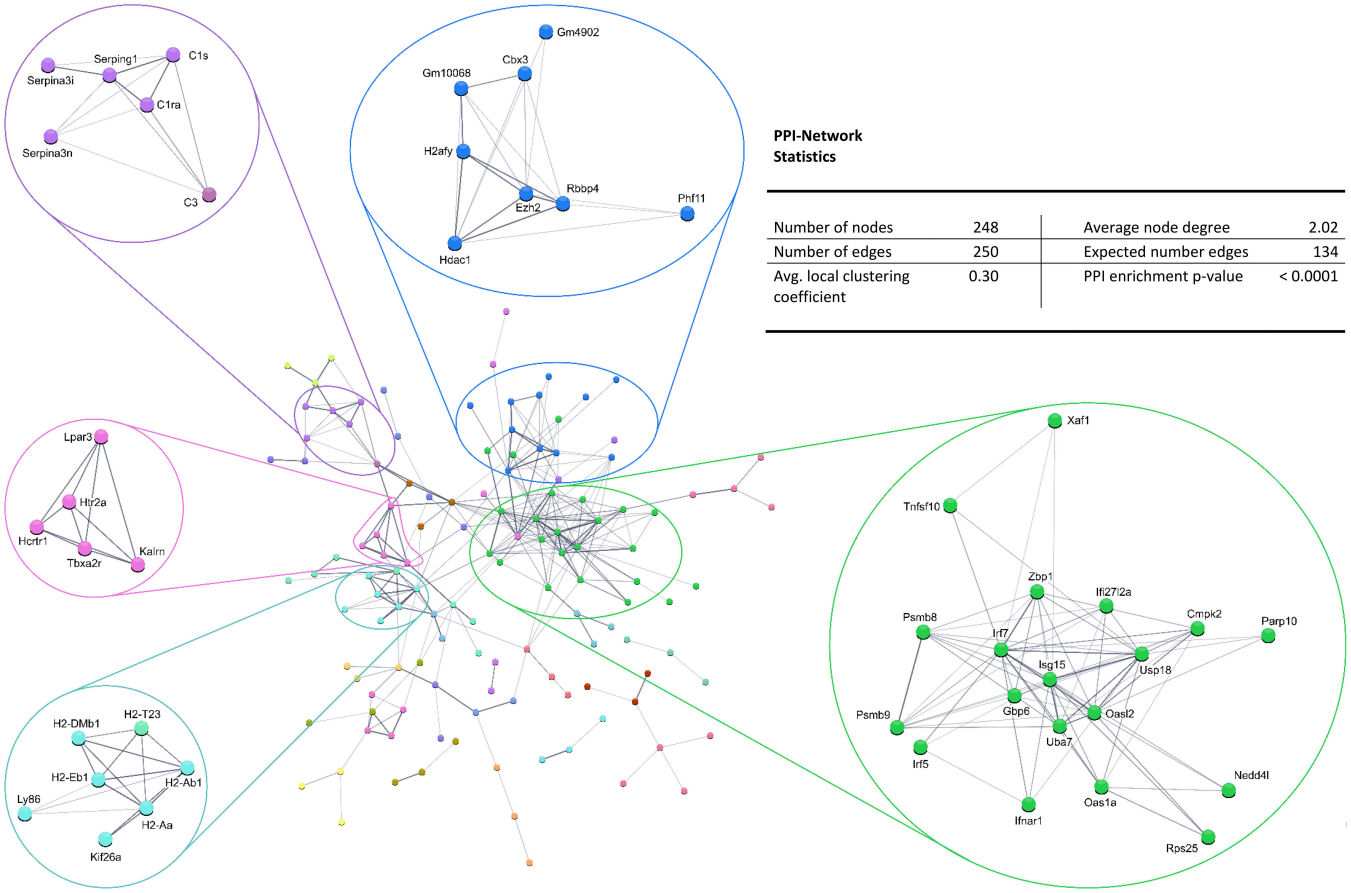
STRING database protein–protein interaction (PPI) network of significantly *downregulated* genes. Cut-off values, *p* ≤ 0.01, fold change ≥ 1.2.

Also, the PPI analysis of significantly downregulated mRNAs revealed a significant PPI enrichment (*p* < 0.0001; Figure 4). Actually observed edges (*n* = 250) exceeded the expected number of edges (*n* = 134) by 87%. Also for the downregulated mRNAs clusters of interconnected proteins emerged. Enrichment analysis showed three clusters (i.e., green, purple, and cyan) related to the immune system (e.g., Immune system process—GO: 0002376, Immune response—GO:0006955). The blue cluster was associated with gene regulation (e.g., Chromatin modification—GO:0016568) and the rose cluster was related to “G-protein coupled receptor activity” (GO:0004930) (Table 6).

**Table 6:** GO biological processes and molecular functions of PPI-clusters from *downregulated* mRNAs in α-Gal A^(−/0)^ vs. wildtype mice.

**Pathway ID**	**Pathway description**	**Count in network**	**False discovery rate**
**GREEN CLUSTER**			
GO:0002376	Immune system process	10	<0.0001
GO:0051707	Response to other organism	8	<0.0001
GO:0009615	Response to virus	7	<0.0001
GO:0002252	Immune effector process	7	<0.0001
GO:0051607	Defense response to virus	6	<0.0001
GO:0045087	Innate immune response	6	0.0005
**BLUE CLUSTER**			
GO:0016568	Chromatin modification	5	0.0007
GO:0045814	Negative regulation of gene expression, epigenetic	4	<0.0001
**PURPLE CLUSTER**			
GO:0006952	Defense response	5	0.0003
GO:0002455	Humoral immune response mediated by circulating immunoglobulin	4	<0.0001
GO:0006958	Complement activation, classical pathway	4	<0.0001
GO:0045087	Innate immune response	4	0.0007
**ROSE CLUSTER**			
GO:0004930	G-protein coupled receptor activity	4	0.0049
**CYAN CLUSTER**			
GO:0006955	Immune response	6	<0.0001
GO:0048002	Antigen processing and presentation of peptide antigen	5	<0.0001
GO:0019886	Antigen processing and presentation of exogenous peptide antigen via MHC class II	4	<0.0001
GO:0003823	Antigen binding	4	<0.0001
GO:0034341	Response to interferon-gamma	3	0.0004
GO:0042605	Peptide antigen binding	3	<0.0001

### Ion channel regulation

As the sensory deficits of Fabry patients are generally accepted to be caused by changes in the excitability of sensory neurons, we specifically searched our dataset for genes related to ion channels, ion channel function and trafficking. Besides downregulation of voltage-gated sodium and calcium channels (i.e., Scn7a and Cacna1h), we found that several potassium channels and potassium channel associated proteins were differentially expressed (Table 7). Voltage-gated (i.e., Kcnb2) and calcium activated potassium channel subunits (i.e., Kcnmb1 and Kcnt1), as well as potassium channel tetramerization and interacting proteins (i.e., Kcnip2, Pctd16, and Kctd11) were downregulated in DRGs from FD mice. In contrast, the potassium channel ancillary beta subunit Kcne3 was upregulated. Last but not least, the mechanosensitive ion channel Piezo2 was significantly downregulated. Against all expectation, we found none of the pain-associated transient receptor potential (TRP) channels regulated.

**Table 7:** Raw expression values, fold changes, and statistical analysis for significantly regulated ion channels.

**NCBI RefSeq ID**	**Gene symbol**	**Gene name**	**Expression α-Gal A^(−/0)^**	**Expression wildtype**	**Fold change**	***p*-value**	**FDR**
**UPREGULATED GENES**							
NM_020574	Kcne3	Potassium voltage-gated channel, Isk-related subfamily, gene 3	160	77	2.0	0.0001	0.0332
NM_001190870	Kcne3	Potassium voltage-gated channel, Isk-related subfamily, gene 3	142	77	1.8	0.0001	0.0236
NM_001042489	Hvcn1	Hydrogen voltage-gated channel 1	215	133	1.6	0.0045	0.1545
NM_146037	Kcnk13	Potassium channel, subfamily K, member 13	1,721	1,049	1.5	0.0000	0.0122
NM_001042489	Hvcn1	Hydrogen voltage-gated channel 1	312	217	1.4	0.0046	0.1565
**DOWNREGULATED GENES**							
NM_031169	Kcnmb1	Potassium large conductance calcium-activated channel, subfamily M, beta member 1	1,759	2,621	−1.6	0.0000	0.0059
NM_011028	P2rx6	Purinergic receptor P2X, ligand-gated ion channel, 6	523	682	−1.4	0.0045	0.1546
NM_175462	Kcnt1	Potassium channel, subfamily T, member 1	11,165	13,232	−1.3	0.0056	0.1701
NM_145703	Kcnip2	Kv channel-interacting protein 2	1,321	1,598	−1.3	0.0027	0.1273
NM_026135	Kctd16	Potassium channel tetramerisation domain containing 16	947	1,140	−1.3	0.0029	0.1327
NM_001039485	Piezo2	Piezo-type mechanosensitive ion channel component 2	1,089	1,258	−1.3	0.0085	0.1956
NM_021415	Cacna1h	Calcium channel, voltage-dependent, T type, alpha 1H subunit	3,547	4,085	−1.3	0.0029	0.1327
NM_009135	Scn7a	Sodium channel, voltage-gated, type VII, alpha	1,193	1,366	−1.2	0.0071	0.1837
NM_001098528	Kcnb2	Potassium voltage gated channel, Shab-related subfamily, member 2	1,600	1,799	−1.2	0.0061	0.1744
NM_153143	Kctd11	Potassium channel tetramerisation domain containing 11	1,445	1,624	−1.2	0.0051	0.1637

### RT-qPCR validation of regulated genes

**Figure 5:**
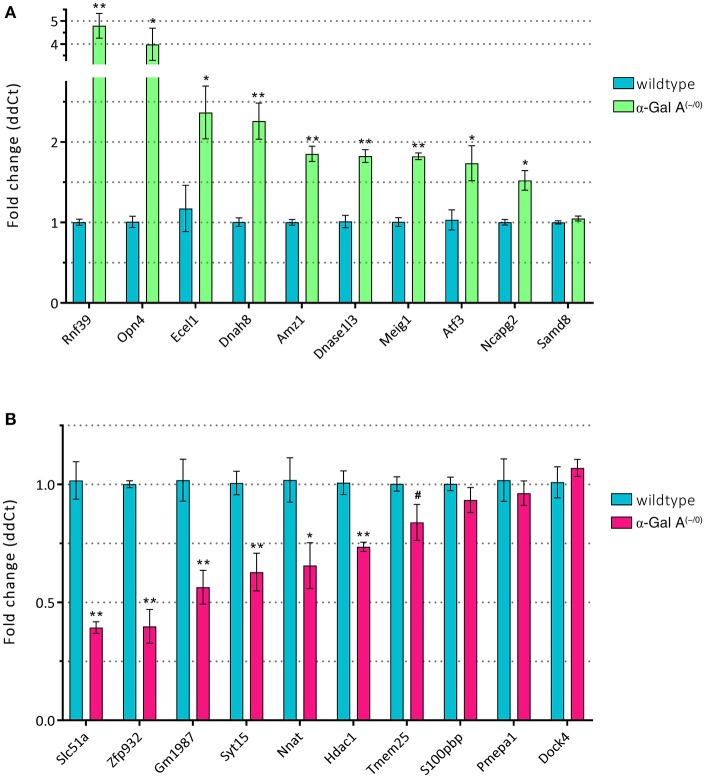
RT-qPCR validation of up- **(A)** and *downregulated* genes **(B)**. ^*^p < 0.05, ^**^p < 0.01, #*p* < 0.1.

To validate the differentially expressed genes from the microarray expression profiling, we performed RT-qPCR analysis of the top 10 up- and downregulated genes in a separate set of samples from α-Gal A^(−/0)^ mice backcrossed with C57BL/6J mice and C57BL/6J wildtype mice. We found that 9/10 of the upregulated genes (i.e., Rnf39, Opn4, Ecel1, Dnah8, Amz1, Dnase1l3, Meig1, Atf3, and Ncapg2) showed significant upregulation, whereas only one gene (i.e., Samd8) was not regulated (Figure 5A). For the downregulated genes, 6/10 genes (i.e., Slc51a, Zfp932, Gm1987, Syt15, Nnat, and Hdac1) were significantly downregulated, and four genes (i.e., Tmem25, S100pbp, Pmepa1, and Dock4) did not show regulation (Figure 5B). Thus, differential expression of 75% of the genes selected for RT-qPCR validation could be verified.

## Discussion

Neuropathic pain and small-nerve fiber neuropathy are among the first symptoms of Fabry disease and affect the majority of patients already in early childhood. Therefore, the involvement of sensory neurons, whose cell somata are located in DRGs, is evident. However, our study is the first to present a differential gene expression profile of DRGs from α-Gal A^(−/0)^ mice, a recognized mouse model for FD (Ohshima et al., 1997; Lakoma et al., 2014; Uceyler et al., 2016), and wildtype controls. We performed microarray expression profiling and found that 812 genes were significantly deregulated, 506 of them being upregulated and 306 being downregulated. Enrichment analysis revealed that the two pathways “lysosome” and “ceramide metabolic process” were significantly enriched. As FD is part of the broad family of lysosomal storage disorders that all show defects in ceramide metabolism (Platt et al., 2012), our results demonstrate the involvement of these two pathways also in DRG neurons and therefore enhance the reliability of the current analysis.

When taking a closer look at the significantly downregulated genes the “immune system” emerged as another disease specific entity. Lysosomal storage disorders in general are associated with deficits in processing of protein antigens and antibody production (Daly et al., 2000), and Hawkins-Salsbury et al. (2011) specifically report an immune deficit in Fabry patients. In the present study, enrichment analysis of downregulated genes revealed mainly immune system related pathways and processes, for example different autoimmune diseases, infection pathways and processes like “immune responses” or “antigen processing and presentation” (Table 5). In this regard, it might be noted that the downregulated purple cluster which includes serine-protease inhibitors (Serpins) might also be involved in nervous system related symptoms. Serpins are known to play a role in coagulation, and loss of serpins might induce a variety of bleeding disorders (Kaiserman et al., 2006). It has recently been shown that angiokeratoma, one of the first dermatologic disease presentations in Fabry patients, if present in gastrointestinal mucosa can lead to life-threatening bleeding episodes during coagulation therapy (Oh et al., 2016; Kang et al., 2017). Interestingly, 30% of Fabry patients show cerebral microbleeds (Kono et al., 2016), which together with the downregulated Thrombospondin 1 (Thsd7a) and Thromboxane a2 receptor (Tbxa2r) can be related to a general deficit in blood coagulation pathways. Further analysis of the regulation and impairment of those genes might open up new treatment strategies for cerebral vasculopathy, including cerebral hemorrhage, stroke, or other cerebral lesions associated with FD (Schiffmann and Moore, 2006). In a mouse model of neuropathic pain, it has been shown that mice that underwent surgery for chronic constriction injury showed activation of the immune system in higher brain structures (Koks et al., 2008). Based on these results it would be interesting to see if this immune activation is also present in brains of FD mice and/or patients.

Enrichment analysis of the upregulated clusters revealed significant enrichment of the “G-protein coupled receptor signaling” and “retrograde transport” pathways. Upregulation of genes in these clusters could be related to hypersensitivity and changes in excitability of DRG nerve fibers as a possible underlying cause of the frequent pain attacks experienced by Fabry patients (Schiffmann and Moore, 2006; Uceyler et al., 2014). Although the genes in the reported clusters are not directly related to changes in excitability, a number of ion channels were significantly deregulated and could be responsible for the hyperexcitability. Besides downregulation of voltage-gated sodium and calcium channels, different potassium channels and associated proteins showed regulation. In contrast, only Kcne3—a potassium channel ancillary beta subunit known to increase excitability (Abbott et al., 2001)—was upregulated. To date, knowledge on changes in ion channel expression and function in FD are sparse and controversial. Lakoma et al. (2014) reported increased immunoreactivity for a voltage-gated sodium channel Na_v_1.8 (Scn10a) in skin samples of FD mouse sensory neurons including the free nerve endings. Recently, decreased conductance of sodium currents in dissociated DRG neurons from FD mice was demonstrated (Namer et al., 2017). This latter publication also reported activation of voltage-gated potassium channels at more depolarized potentials, supporting a general reduction in FD neuron excitability (Namer et al., 2017). With regard to calcium channels it has been shown that Lyso-Gb3 enhances voltage-gated calcium currents in DRGs of FD mice (Choi L. et al., 2015), whereas Namer et al. (2017) report decreased voltage-gated calcium currents in α-Gal A^(−/0)^ nociceptors. We also found a downregulation of the mechanosensitive ion channel Piezo2 mRNA, which may possibly be correlated to the decreased number of mechanosensitive fibers found in both human patients and FD mice (Namer et al., 2017). With regards to temperature sensitive ion channels it has been shown that expression of Trpv1 was increased, whereas expression of Trpm8 was decreased in skin biopsies of FD mice (Lakoma et al., 2014, 2016), which may be related to the changed thermal thresholds reported in both Fabry patients and mice (Sheth and Swick, 1980; Dutsch et al., 2002; Uceyler et al., 2013; Namer et al., 2017). The present unbiased screen for differentially expressed ion channels did not confirm deregulation of Trpv1 or Trpm8 though.

Previous gene expression studies were not performed in neuronal tissues but could still be affected by the same regulating pathways. In α-Gal A^(−/0)^ fibroblasts and endothelial cells K_Ca_3.1 (Kcnn4) was downregulated (Choi et al., 2014; Choi J. Y. et al., 2015) and the conductance of calcium-activated potassium channels was reduced (Olivan-Viguera et al., 2017). Additional gene expression studies have been performed in hepatic, renal and human blood cells (Park et al., 2009; Cigna et al., 2013; Shin et al., 2015). Thrombospondin 2 and 4 have been found to be upregulated in FD kidney cells (Park et al., 2009), whereas our results show a downregulation of both Thrombospondin 1 (Thsd7a) and Thromboxane a2 receptor (Tbxa2r) in FD DRGs. Both observations point towards impaired blood coagulation pathways in FD. In the same screen Neuropeptide Y (NPY) was found to be upregulated (Park et al., 2009). In the current dataset, a different neuropeptide, Neuropeptide B, was significantly upregulated, which has been shown to be functionally connected with NPY at least in fish (Yang et al., 2014). Furthermore, different types of S100 calcium binding proteins, i.e., S100a4/a8/a9 are upregulated in liver and kidney (Park et al., 2009), whereas S100pbp (a S100P binding protein) was decreased in FD DRGs in the present screen.

The deregulated genes that emerged from our analysis largely overlap with genes from previous reports on other painful disorders, although the direction of regulation does not always match. Upregulation of the transcription factor Atf3 is in line with previous reports that showed induction of Atf3 in DRGs in different models of nerve injury (Tsujino et al., 2000; Shortland et al., 2006; Matsuura et al., 2013), as well as upon exposure to noxious stimuli (Braz and Basbaum, 2010). Also, the adenosine receptor Adora2b which was upregulated in FD mice promotes chronic pain through neuro-immune interactions (Hu et al., 2016). The Tyrp1 gene has been associated with thermal nociception, and loss of function mutations generate deficits in thermal nociception (Fortin et al., 2010). Furthermore, Cdk5-mediated phosphorylation modulates Trpv1 function (Jendryke et al., 2016). Upregulation of these two latter genes in FD may therefore be associated with burning and tingling paraesthesias reported in Fabry patients (Germain, 2010; Ginsberg, 2013). Neuronatin (Nnat), which is significantly downregulated in the current screen, was upregulated in DRGs after sciatic nerve injury and associated with mechanical hypersensitivity (Chen et al., 2010). Several genes in the clusters that emerged from the current analysis, are associated with G-protein signaling and are controversially discussed (Pan et al., 2008). The somatostatin receptor Sstr2 in the red cluster is downregulated after sciatic nerve ligation (Shi et al., 2014), but elevated in response to intestinal inflammation (Van Op den Bosch et al., 2009). The endothelin receptor Ednrb attenuates cancer-induced pain (Viet et al., 2011), and the angiotensin receptor Agtr1b has been proposed as a biomarker for pain (Grace et al., 2012). All clusters involve genes that have been associated with exacerbated pain phenotypes in clinical or preclinical studies. Single nucleotide polymorphisms in the serotonin receptor gene Htr2a in the rose gene cluster are associated with pain-phenotypes as a genetic predisposition to musculoskeletal pain (Nicholl et al., 2011). The hypocretin receptor Hcrtr1 is associated with migraine (Rainero et al., 2011), and Kalirin (Klrn), a Rho guanine nucleotide exchange factor, is required for persistent nociceptive activity dependent synaptic long-term potentiation (Lu et al., 2015). The pink cluster contains genes that are mainly associated with retrograde transport. Vps26a is increased following spinal nerve ligation in the spinal dorsal horn and is required for recycling of mGluR5 and plasticity at excitatory synapses (Lin et al., 2015). Vps35, another regulated gene product from our screen, forms a complex with Vps26a (Kim et al., 2010) and is also highly associated with members from the sorting nexin family (e.g., Snx6 and Snx8 from our screen). Individuals with polymorphisms in Gluthatione-S-transferase genes found in the orange gene cluster are more likely to develop neuropathy during oxaliplatin treatment (Kanat et al., 2017). In addition, activation of Aldh2, a gene associated with the glutathione pathway, reduces nociception in acute inflammatory pain (Zambelli et al., 2014). This gene is regulated by Aldh3a1 which was deregulated in the current analysis (Chen et al., 2015). The green cluster contained the gene Tnfsf10, a member of the Tumor necrosis factor superfamily. Tnfsf10 is increased by excitotoxic spinal cord injury (Plunkett et al., 2001), downregulated in inflamed tissue (Yang et al., 2007), and associated with migraine susceptibility (Jia et al., 2015). Parp10, a poly(ADP-ribose) polymerase upregulates pro-inflammatory pathways, and its inhibition attenuates neuropathy and neuroinflammation (Komirishetty et al., 2016a,b). Interferon regulatory factor Irf5 is increased in spinal microglia after peripheral nerve injury and drives P2X4R+ reactive microglia thereby gating neuropathic pain (Masuda et al., 2014). In Chronic atypical neutrophilic dermatosis with lipodystrophy and elevated temperature (CANDLE), a disease exhibiting joint pain symptoms, mutations have been found in proteasome subunit genes Psmb8 and Psmb9 (Arimochi et al., 2016). Genes from the Oas dsRNA sensor family, in particular Oas1a and Oasl2, are induced by lipopolysaccharides, which induce inflammatory pain (Lee et al., 2013). The E3 ubiquitin ligase Nedd4 is decreased in DRGs of SNI mice (Laedermann et al., 2013), and ribosomal protein Rps25, as well as other ribosomal proteins are downregulated in a model for HIV-associated neuropathic pain (Maratou et al., 2009). In line with the downregulation of Hdac1 in the blue cluster, HDAC inhibitors attenuate the development of hypersensitivity (Denk et al., 2013), restore C-fiber sensitivity (Matsushita et al., 2013), and induce behavioral anti-nociception (Tao et al., 2016). In addition, nerve injury increases the activity of Hdac1 and Ezh2 (Laumet et al., 2015). Pain responses depend on genes from the major histocompatibility complex (MHC; Guo et al., 2015), and the MHC-2 haplotype is involved in the incidence of postherpetic pain (Sato-Takeda et al., 2006). Further, MHC-2 molecules synergize with Toll-like receptor Tlr4 in inducing an innate immune response (Frei et al., 2010), and the lymphocyte antigen Ly86 is required in DRG neurons for functional Toll-like receptor Tlr4 signaling (Grace et al., 2014). Serpina3n is upregulated in mouse DRGs following nerve injury and attenuates neuropathic pain. Mice lacking Serpina3n develop more severe neuropathic pain symptoms than wildtypes (Vicuna et al., 2015). Another member of the Serpin family—Serping—has been implicated in hereditary angioedema, as mutations in this gene are associated with abdominal pain symptoms (Andrejevic et al., 2015). Finally, the Complement component genes C1r, C1s and C3 are upregulated after spinal nerve ligation (Levin et al., 2008). When comparing the emerging FD pain related genes with the global pain systems network for heat nociception (Neely et al., 2012) only epidermal growth factor receptor pathway substrate 8 (Eps8), alpha-N-acetylgalactosaminidase (Naga) and the proteasome subunit gene Psmb8 were contained. Together, this comprehensive literature search demonstrates considerable overlap of the current FD expression profile with genes implicated in nociception and pain disorders, suggesting relevant common pathogenesis components of FD pain and other pain disorders.

Despite constituting the first presentation of differentially expressed genes in DRG explants of α-Gal A^(−/0)^ mice, some limitations of the current analysis need to be considered. For instance, concerns have been raised that the α-Gal A^(−/0)^ mouse model might only resemble the later-onset phenotype of FD (Bangari et al., 2015). In kidney, Gb3 concentrations only reach 25% of that found in patients, and FD mouse life expectancy is normal (Taguchi et al., 2013). Therefore, the G3Stg/GLA knockout mouse has been generated and evaluated as a new FD mouse model, in which α-Gal A^(−/0)^ mice were crossbred with transgenic mice expressing the human Gb3 synthase. This resulted in symptomatic animals with increased Gb3 accumulation and progressive renal impairment (Taguchi et al., 2013). Another FD mouse model is the NOD/SCID immune deficiency mouse model with tissue specific Gb3 accumulation, but without clinical manifestation (Pacienza et al., 2012). Few data are available for these genetic models yet, but it would be important to know to what extent the three FD mouse models share the same differential gene expression. In addition, it would be of interest to explore the deregulation of gene expression in heterozygote females, which in humans and mice exhibit a considerably weaker phenotype than males (Uceyler et al., 2013, 2016). Screening of homozygote females could be helpful to better understand the mechanisms and degree of X-chromosomal inactivation in female Fabry patients (Wilcox et al., 2008). Finally, it should be noted that gene targeting experiments are prone to a general phenomenon of background dependence that might confound the interpretation of results (Schalkwyk et al., 2007). In this study we controlled for this effect by using α-Gal A^(−/0)^ mice that had been backcrossed to an inbred C57BL/6J colony for the RT-qPCR validation of regulated genes.

Our in-depth bioinformatics analysis revealed a new set of genes and pathways that might be involved in the FD-associated small-nerve fiber neuropathy. These data give rise to subsequent functional studies on the importance of these deregulated genes for the pathogenesis of FD small fiber disease and neuropathic pain, and are expected to lead to the identification of novel treatment strategies, especially for neuropathic pain related symptoms in Fabry patients.

## Author contributions

KK, MK, and ML: designed the study; KK, TK, and ML: performed the data collection, analyzed, and interpreted the data; KK: wrote the manuscript. TK, MK, and ML: critically reviewed the contents of the paper and suggested substantial improvements; All authors have approved the final version of the manuscript.

### Conflict of interest statement

The authors declare that the research was conducted in the absence of any commercial or financial relationships that could be construed as a potential conflict of interest.
